# Posterior-only surgical correction of adolescent idiopathic scoliosis: an Egyptian experience

**DOI:** 10.1051/sicotj/2017057

**Published:** 2017-12-11

**Authors:** Belal Elnady, Mohammad M. El-Sharkawi, Mohamed El-Meshtawy, Faisal F. Adam, Galal Zaki Said

**Affiliations:** Department of Orthopedic and Trauma Surgery, Assiut University Medical School, 71111 Assiut Egypt

**Keywords:** All pedicle screws, Idiopathic scoliosis, Adolescent spine deformity.

## Abstract

*Introduction*: The aim of this prospective case series study is to document safety and effectiveness of high density pedicle screws through posterior only approach with intraoperative wake-up test in correction of adolescent idiopathic scoliosis (AIS).

*Methods*: Between 2011 and 2015, all surgically treated patients for AIS were followed up for a minimum of 2 years. Clinical outcomes were evaluated using scoliosis research society-22 (SRS) questionnaire. All patients were classified according to Lenke classification. Major and minor curves Cobb angle as well as sagittal parameters were measured on whole spine X-rays. All patients underwent an intra-operative wake-up test after deformity correction and a minimum of 80% metal density of implants was used.

*Results*: This study included 50 patients. The mean age at time of surgery was 16.8 years. The mean follow-up period was 38.1 months. The mean correction rate for the coronal Cobb angle of the major curve was 79.12%, while that of the minor curve was 68.9%. The mean thoracic kyphosis angle was 38.4° preoperatively, 29.76° postoperatively and 30.36° at the last follow-up. The mean SRS-22 questionnaire scores improved significantly at the last follow-up (*P *> 0.001). There were no neurological deficits at the wake-up test. No cases of pseudarthrosis or metal failure were encountered.

*Conclusion*: This is a prospective study of at least 80% metal density pedicle screws technique and intra-operative wake-up test in Egyptian patients with AIS. It proved to be an effective and safe technique in correction of radiological parameters, with no neurological or implant related complications. It allowed excellent scoliotic and kyphotic curves correction with minimal loss of correction. On the whole it led to better quality of life.

## Introduction

The aim of surgical correction of adolescent idiopathic scoliosis (AIS) is to achieve a balanced spine, maintain normal neurological function and preserve as many motion segments as possible. This is performed by fusion of the spine following correction of the deformity. Deformity correction may be performed through an anterior open or thoracoscopic approaches, a posterior approach or combined approaches [1]. All these approaches have their specific advantages and disadvantages.

The optimal implants for posterior fixation should achieve better curve correction with high safety profile and minimal implant related complications. Hooks, hybrid constructs and all pedicle screws have been extensively used for posterior correction of AIS [2]. Pedicle screws, by allowing secure segmental three-column fixation through the familiar posterior approach, have become the principle method for scoliosis correction with many advantages over other fixation methods [3].

Neurological deficits are the second most feared complication in scoliosis surgery after death. Trials to avoid neurological injuries during scoliosis correction started since a long time with wake-up test, ankle clonus test, and recently with somatosensory evoked potential and transcranial motor stimulation [4].

The aim of this study was to evaluate the efficacy and safety of using high density all pedicle screws with intraoperative wake-up test to correct spinal deformity due to AIS in Egyptian patients. To the best of our knowledge, this is the first prospective cohort series using this technique in our locality.

## Patients and methods

Between 2011 and 2015, fifty patients surgically treated for AIS were prospectively followed for a minimum of 2 years. This study was approved by our institution ethical committee.

Inclusion criteria: Patients, attending outpatient spine clinic of our hospital, with AIS older than 10 years of age with curves more than 45°.

After a thorough clinical evaluation, preoperative whole spine erect anteroposterior (AP), lateral and side bending X-rays were made for every patient.

On the AP view films, Cobb angle of the major curve as well as the minor curve was measured. Global coronal balance was measured as the distance between C7 plumb line and central sacral vertical line (CSVL). On the lateral view films, thoracic kyphosis was measured from T5 upper end plate (or higher if visible) to T12 lower end plate and lumbar lordosis was measured from L1 upper end plate to S1 upper end plate. Global sagittal balance was assessed by the distance between C7 plumb line and posterosuperior corner of S1 with positive values in front and negative values behind the posterosuperior corner of S1. The Cobb angle was also measured on the side bending films to assess curves' flexibility [5].

All patients were classified according to Lenke et al. classification [6] into one of the six main types. Lumbar and thoracic modifiers were determined for every patient and only structural curves were fused. Selective thoracic fusion was done for patients with Lenke type 1 with lumbar modifiers A or B.

The upper instrumented vertebra was T4 if the patient had high right shoulder, T3 if the patient had balanced shoulders and T2 if the left shoulder was high. In Lenke 5 with lumbar curve, fusion stopped at T10 or T9. The lower instrumented vertebra was the end vertebra if it was touched by the CSVL and end+1 or end+2 if the end vertebra was not touched by CSVL.

All patients were operated in the prone position on a transparent surgical table under general anesthesia. Pedicle screws were inserted in more than 80% of pedicles across the instrumented levels using the free hand technique. Extended head screws were used as a routine on concave side to assist in apical reduction. After finishing pedicle screws insertion, their optimal position was checked using intraoperative fluoroscopy. Bilateral facetectomy across the fusion levels was routinely done to improve curve flexibility and enhance fusion. Additional release procedures were done in selected cases with rigid curves and this ranged from multiple Ponte osteotomies to asymmetrical pedicle subtraction osteotomy (PSO). Concave rod was inserted first and then derotation of the spine was done by rod derotation technique. Insertion of the convex rod, final segmental compression, distraction, and direct vertebral rotation were used to improve correction.

As intra-operative neuromonitoring is not available in our hospital, the Stagnara wake-up test [7] was performed immediately after inserting the second rod in every patient to ensure neural integrity. Posterior fusion was then done using autologous local bone graft. In this series, no iliac bone graft was used, no local antibiotic was added to the bone graft, no costoplasty was done and Tranexamic acid was not used as a routine. Finally, the wound was closed in layers over a suction drain.

All patients were mobilized as early as they tolerated without any external support. The preoperative radiological parameters were re-measured immediately after surgery and at each follow up visit at 2, 6, 12 months postoperatively and at yearly interval thereafter. Functional outcome was assessed using the SRS-22 questionnaire. Statistical analysis was done using the SPSS program (IBM SPSS version 20.0) using chi-square and *t*-test. *P* value less than 0.05 is considered statistically significant.

## Results

This study included 50 patients, 39 females (78%) and 11 males (22%). The mean age at surgery was 16.8 years (range, 11–23 years). The mean follow up period was 38.1 months (range, 28–64 months).

The mean operative time was 260 min (range, 180–440 min), with mean blood loss of 1100 cc (range, 400–1900 cc), and average 3 units (range, 1–6 units) of blood transfusion were needed. The average number of fusion levels was 12.3, with a mean of 21.3 pedicle screws inserted per patient, with total 1063 screws inserted in 617 levels. The average implant density was 86.6% (21.3 screws/24.6 pedicles). The lowest instrumented vertebra was the end vertebra in 14 cases, end+1 in 19 cases and end+2 in 17 cases. Ponte osteotomy was done in 28 patients. Five patients required asymmetrical PSO due to very rigid curve.

**Figure 1:**
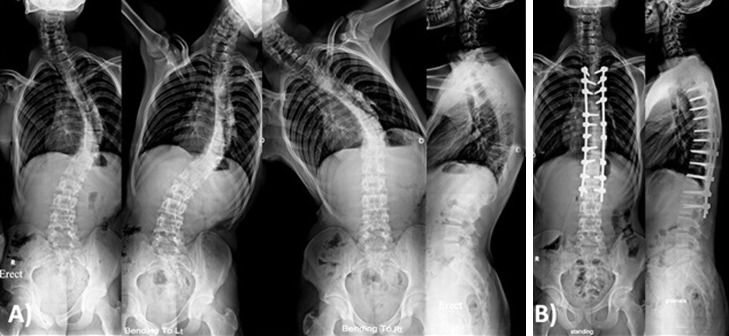
Male patient 17 years old with Lenke 1 a n. (A) Preoperative X-rays showing main thoracic curve of 50° that was corrected to 29° in side bending films with thoracic kyphosis of 40°. (B) One year postoperative X-rays showing posterior instrumentation from T4-L2 with main thoracic curve correction to 9° with thoracic kyphosis of 36°.

**Figure 2:**
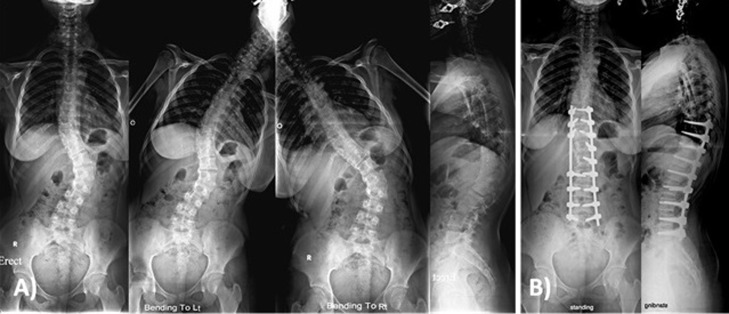
Female patient 16 years old with Lenke 5 c +. (A) Preoperative X-rays showing lumbar curve of 56° that was corrected to 41° and main thoracic curve of 28° that was corrected to 20° in side bending films, with thoracic kyphosis of 48°. (B) Two years postoperative X-rays showing posterior correction and fusion of the lumbar curve only from T9-L5 with lumbar curve correction to 10° and spontaneous main thoracic curve correction to 14° with thoracic kyphosis of 38°.

**Figure 3:**
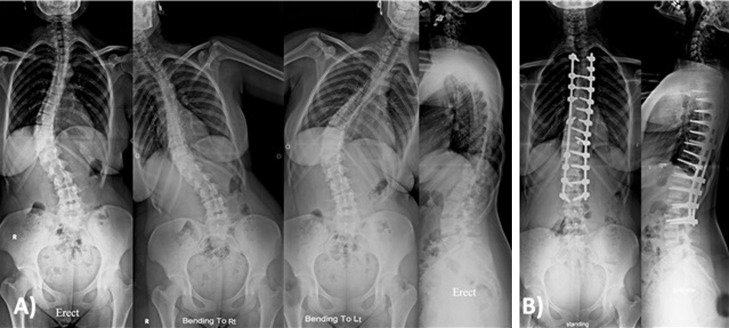
Female patient 18 years old with Lenke 1 c n. (A) Preoperative X-rays showing main thoracic curve of 55° that was corrected to 15° in side bending films with thoracic kyphosis of 38°. (B) Two years postoperative X-rays showing posterior correction and fusion from T4-L3 with main thoracic curve correction to 5° with thoracic kyphosis of 26°.

The most common curve type was type I (Figures 1 and 3) in 24 patients, followed by type V (Figure 2) in 11 patients (Table 1). Thoracic kyphosis was normal in 27 patients (Sagittal modifier N), hypokyphosis in 4 patients (Sagittal modifier −) and hyperkyphosis in 19 patients (Sagittal modifier +).

**Table 1:** Classification according to Lenke.

Lenke type	Total (%)	−	*N*	+
I Main thoracic	24 (48%)	1	14	9
II Double thoracic	1 (2%)	–	–	1
III Double major	8 (16%)	–	5	3
IV Triple major	2 (4%)	–	1	1
V Thoracolumbar/lumbar	11 (22%)	1	5	5
VI Thoracolumbar/lumbar with main thoracic	4 (8%)	2	2	–

The mean major curve Cobb angle was 61.3° preoperatively, with a mean curve flexibility of 29% (Table 2). This was corrected to 12.8° postoperatively (*P* < 0.001), and to 13.5° at last follow up. The percent of curve correction was 79.1% with 1.44% loss of correction at last follow up (*P* = 0.689).

**Table 2:** Coronal parameters.

	Preop.	Postop.	*P* value 1	Final visit	*P* value 2	Correction %	% loss
Main curve Cobb angle	61.33 ± 15.4°	12.78 ± 8.84°	<0.001**	13.51 ± 9.35°	0.689	79.12% ± 9.61	1.44% ± 2.2
Minor curve Cobb angle	38.16 ± 15.4°	11.87 ± 11.09°	<0.001**	12.86 ± 9.62°	0.546	68.89% ± 20.07	3.765% ± 5.42
Coronal balance	2.26 ± 1.46 cm	1.34 ± 1.44 cm	0.003**	1.06 ± 0.99	0.112	–	–

P value 1: between preoperative and postoperative.

P value 2: between postoperative and last follow up.

** Highly significant difference (*P* < 0.01).

The mean minor curve Cobb angle was 38.16° preoperatively (Table 2) has been corrected to a mean of 11.87° postoperatively (*P* < 0.001) and was 12.86° at last follow up with 68.9% percentage correction of the minor curve and 3.77% percentage loss of correction at last follow up (*P* = 0.546). The mean global coronal balance was 2.26 cm preoperatively, 1.35 cm postoperatively (*P* = 0.003) and 1.05 cm at last follow up (*P* = 0.112).

The mean thoracic kyphosis (Table 3) was 38.44° preoperatively, 29.76° postoperatively (*P* < 0.001), and 30.36° at the last follow up (*P* = 0.264). The mean lumbar lordosis was 47.62° preoperatively, 40.09° postoperatively (*P* > 0.001) and 41.2° at last follow up (*P* = 0.135). The mean global sagittal balance was (−) 8.5 mm preoperatively and (+) 3.7 mm postoperatively (*P* = 0.032) and (+) 2.7 mm at last follow up (*P* = 0.608).

**Table 3:** Sagittal parameters.

	Preop.	Postop.	*P* value 1	Final visit	*P* value 2
Thoracic kyphosis	38.44 ± 16.71°	29.76 ± 11.05°	<0.001**	30.36 ± 11.03°	0.264
Lumbar lordosis	47.62 ± 14.64°	40.09 ± 12.1°	<0.001**	41.2 ± 10.62°	0.135
Global sagittal balance	−8.5 ± 27.8 mm	+3.7 ± 27.5 mm	0.032*	+2.7 ± 23.1 mm	0.608

*P* value 1: between preoperative and postoperative.

*P* value 2: between postoperative and last follow up.

* Significant difference (*P* < 0.05).

** Highly Significant difference (*P* < 0.01).

The SRS-22 questionnaire improved significantly from a mean of 2.77 preoperative to 3.68 at last follow up (*P* < 0.001). Both total score and 5 domains scores improved after surgery.

Intra-operatively, there were no “positive” wake-up tests and no cases with neurological deficits post-operatively. No metal failure or pseudarthrosis was detected during the follow up period. There was one case with wound infection that required debridement surgery without implant removal. At 1 year follow up, there was no evidence of recurrence of infection. One patient with asymmetrical PSO developed hemothorax postoperatively that was managed with chest tube insertion for 24 h.

**Figure 4:**
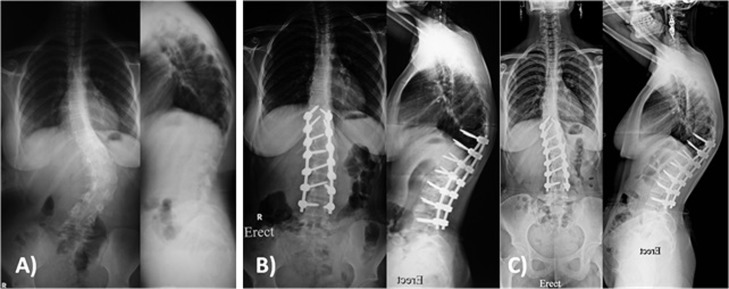
Female patient 17 years old with Lenke 5 c +. (A) Preoperative X-rays showing lumbar curve of 56° with thoracic kyphosis of 50°. (B) Postoperative X-rays showing posterior correction and fusion from T10-L4 with lumbar curve correction to 9° with thoracic kyphosis of 53° and radiological PJK. (C) Two years follow-up X-rays showing no progression of the PJK with preserved coronal correction.

Asymptomatic proximal junctional kyphosis (PJK) was observed in 2 patients (Figure 4). Radiological adding-on for the non-instrumented proximal thoracic and distal lumbar curve was observed in 3 patients with Lenke type 1, for whom selective thoracic fusion had been done. However these patients were asymptomatic and no further surgical intervention was required.

## Discussion

In this prospective cohort study in Egyptian patients with AIS, 79.1% coronal correction of the major curve and 68.9% of the minor curves was achieved. The global coronal balance was 1.35 cm postoperatively and 1.05 at final follow up. These results indicate satisfactory balanced spine after correction. This 79.1% correction rate is higher than most of the previously reported studies using all pedicle screws, which ranged between 62% and 77% [8–12]. In this study, increasing implant density as well as using extended head reduction screws on the concave side offered an efficient derotation maneuver and have resulted in better curve correction. Also, the routine facetectomy in all patients and Ponte osteotomy or even asymmetrical PSO in rigid curves have contributed to improved coronal curve correction.

In the sagittal profile, the mean final thoracic kyphosis in this series is 30.4° and the mean lumbar lordosis is 41.2°. This falls within the normal range of thoracic kyphosis and lumbar lordosis and indicates the ability of all pedicle screws through posterior-only approach to maintain normal sagittal alignment. Also, there were 4 patients with preoperative thoracic hypokyphosis [sagittal modifier ‑] with mean preoperative thoracic kyphosis of 2.75° that has been corrected to a mean of 22.29° at last follow up. This indicates the ability of this technique to restore thoracic kyphosis in patients with preoperative hypokyphosis and concurs with the results of the recently published meta-analysis of thoracic kyphosis in AIS patients corrected by pedicle screws or hybrid constructs [13].

Several previous studies confirmed superiority of pedicle screws to hooks or hybrid constructs. All pedicle screws constructs have achieved superior correction in coronal and axial plane when compared to hook or hybrid constructs. The loss of correction was much less in the all screws in comparison to all hooks or hybrid constructs [2,14,15].

The optimal implant density during posterior correction of AIS is still debated in the literature. Thiong et al. studied the number of fixation anchors associated with optimal curve correction in AIS patients. They concluded that increasing implant density more than 70% within the main curve results in better correction in the coronal plane [16].

The Stagnara wake-up test is a simple, cost-effective maneuver and can be done in any operating room without the need for specialized neurophysiologists. It provides direct measurement of global motor function and has been considered the “gold standard” for the intraoperative assessment of neurological function for a long time [7]. Wake-up test proved to be effective and suitable in low income countries. Recent electrophysiological tools for spinal cord monitoring are expensive, operator dependent, significantly affected by the anesthetic agents and may be negatively affected by many elements of the operating room [17].

Pedicle screws related complications can result from initial malposition of the screws or due to screws pull out during deformity correction resulting in neurological, vascular or visceral injury as well as loss of correction in the postoperative period. Despite the high rate of screw misplacement that can reach up to (15.7%) in some studies [18], the incidence of major complication requiring revision surgery is very low and have only been reported as single case reports [19]. Several techniques have been used to guarantee safety of thoracic pedicle screws. These include the anatomical free hand technique [20] as well as the open lamina technique [21] with direct visualization of the medial wall of the pedicle. The anatomical free hand technique was used in this series and no screws related complication requiring revision was encountered out of 1063 screws used.

Few complications occurred in this series; none of them was long lasting. The overall complications rate in this series is comparable to previous reports on surgery for AIS correction [22,23]. One patient (2%) had wound infection that improved after debridement surgery. The SRS reported overall infection rate of 2.1% after spine surgery, 2.8% after AIS surgery in adult patients and 1.4% after AIS surgery in children [24].

Three patients (6%) in this series developed adding-on for the non-instrumented curves without need for further intervention. The incidence of adding-on is variable in the literature. Suk et al. [12] reported 8% after 5 years follow up (17 adding-on out of 203 patients). Matsumoto et al. [25] reported 18.8% (21 adding-on out of 112 patients), while Wang et al. [26] reported 51.1% incidence of distal adding-on after selective thoracic fusion. Although selective fusion carries the risk of adding-on, it avoids very long fusion and maintains the mobility of the lumbar spine.

Two patients (4%) in this series developed radiological PJK. These patients are asymptomatic, which concurs with similar published studies [27]. In his systematic review on PJK after spinal deformity correction, Cho et al. [28] concluded that despite its high prevalence (up to 30%), radiographic PJK, it does not correlate with poor clinical outcome. Proper selection of the upper instrumented vertebrae, restoration of the sagittal balance and careful dissection with avoiding injury of the facet capsule of the non-fused levels can minimize the incidence of PJK [28].

The limitations of our study include relatively short follow up period. However, previous studies using all pedicle screws with longer follow up period showed insignificant loss of correction over time [11,29]. All pedicle screws technique also prevents crankshaft phenomenon in young patients due to their 3 column fixation extending into the anterior column [30].

The posterior approach for scoliosis correction is a simple exposure, familiar to all surgeons, with little risk for major vascular injury and with good control of the coronal and sagittal plane of the spine. Also, it preserves the pulmonary function as the chest cage is not violated under normal circumstances. Olgun and Yazici reviewed the advantages of anterior versus posterior surgery and concluded that with modern pedicle screws, posterior surgery provides most, if not all, of the advantages of anterior surgery without its negative effect on pulmonary function [1].

In conclusion, all pedicle screws technique (with at least 80% metal density) is an effective and safe method for treatment of AIS. It can achieve excellent curve correction with minimal loss of correction. It also restores the sagittal parameters within normal range leading to better sagittal balance. Using the intra-operative wake-up test proved to be efficient and suitable to low income countries. The screw related complications are minimal and major complications rarely occur. This results in improved functional outcomes with more patient satisfaction and better quality of life.

## Conflict of interest

The authors certify that they have no financial conflicts of interest in connection with this article.
